# Early bactericidal activity of sitafloxacin against pulmonary tuberculosis

**DOI:** 10.1128/spectrum.01645-24

**Published:** 2024-12-10

**Authors:** Lihui Nie, Jing Tong, Guihui Wu, Juan Du, Yuanyuan Shang, Yufeng Wang, Zhangjun Wu, Yuanhong Xu, Yi Ren, Youyi Rao, Yu Pang, Mengqiu Gao

**Affiliations:** 1 Department of Bacteriology and Immunology, Beijing Chest Hospital, Capital Medical University/Beijing Tuberculosis & Thoracic Tumor Research Institute, Beijing, China; 2 Department of Tuberculosis, Beijing Chest Hospital, Capital Medical University/Beijing Tuberculosis & Thoracic Tumor Research Institute, Beijing, China; 3 Department of Tuberculosis, Chengdu Public Health Clinical Medical Center, Sichuan, China; 4 Tuberculosis IV Ward, Wuhan Pulmonary Hospital, Hubei, China; 5 Department of Tuberculosis Control Clinical Center, Beijing Chest Hospital, Capital Medical University/Beijing Tuberculosis & Thoracic Tumor Research Institute/Tuberculosis Clinical Center of China Center for Disease Control and Prevention, Beijing, China; National Center for Biological Sciences, Bangalore, Karnataka, India

**Keywords:** tuberculosis, fluoroquinolones, isoniazid, levofloxacin, sitafloxacin, antituberculosis action, early bactericidal activity

## Abstract

Sitafloxacin is a quinolone broad-spectrum antimicrobial agent, and its pharmacologic properties and *in vitro* data demonstrate that sitafloxacin has a potent killing effect against *Mycobacterium tuberculosis*, including drug-resistant strains, which is superior to that of other available quinolones. However, its efficacy in patients with primary-sensitive tuberculosis is unclear. This study aims to evaluate the early bactericidal activity (EBA) of sitafloxacin in patients with primary drug-susceptible tuberculosis. In this early bactericidal activity study, 30 patients with primary smear-positive tuberculosis were randomized to the once-daily oral administration of 200 mg sitafloxacin, 500 mg levofloxacin, or 300 mg isoniazid (INH) for 7 days. Sputum for quantitative culture was collected 2 days before the study of drug administration, followed by 16 hours of overnight sputum collected daily for 7 days of monotherapy. Colony-forming units (CFU) of *Mycobacterium tuberculosis* were counted from the collected overnight sputum on agar plates to calculate the EBA, defined as log_10_ CFU/mL sputum/day. The bactericidal activity was measured by measuring the first 2 days (early bactericidal activity 0–2) and the last 5 days (prolonged early bactericidal properties 2–7) of study drug administration. The EBA 0–2 of INH (0.39 ± 0.22 log_10_CFU/mL/day) was higher than that of levofloxacin (0.26 ± 0.27 log10CFU/mL/day) and sitafloxacin (0.22 ± 0.25 log_10_CFU/mL/day), with no statistically significant difference (*P* = 0.08). EBA 0–2 was similar for the three drugs. INH prolonged early bactericidal activity (2–7) (0.17 ± 0.16 log_10_CFU/mL/day) was higher than levofloxacin (0.14 ± 0.10 log_10_CFU/mL/day) and lower than sitafloxacin (0.26 ± 0.31 log_10_CFU/mL/day), with no statistically significant difference (*P* = 0.59). The EBA 2–7 of sitafloxacin showed higher activity than INH and levofloxacin. Sitafloxacin exhibits comparable early bactericidal activity and higher extended early bactericidal activity relative to levofloxacin. In addition, this novel fluoroquinolone has a good safety profile. The study data highlights the potential of sitafloxacin in the clinical management of drug-susceptible tuberculosis, as well as drug-resistant tuberculosis.

**IMPORTANCE:**

Sitafloxacin is a quinolone broad-spectrum antimicrobial agent, and its pharmacologic properties and *in vitro* data demonstrate that sitafloxacin has a potent killing effect against *Mycobacterium tuberculosis*. However, its efficacy in patients with primary-sensitive tuberculosis is unclear. We investigated the early bactericidal activity of sitafloxacin in primary susceptible tuberculosis. The results showed that sitafloxacin exhibited comparable early bactericidal activity and higher extended early bactericidal activity relative to levofloxacin. In addition, this novel fluoroquinolone has a good safety profile. Our study data highlights the potential of sitafloxacin in the clinical management of drug-susceptible tuberculosis, as well as drug-resistant tuberculosis.

## INTRODUCTION

Tuberculosis (TB) continues to pose a significant global health threat, with 1.6 million deaths reported worldwide in 2021, according to the World Health Organization (WHO) (1). Alarmingly, the incidence of TB is on the rise, accompanied by an increase in mortality rates. In 2021, there were 450,000 new cases of rifampicin-resistant tuberculosis, marking a notable escalation from the previous year. However, the success rate of treating drug-resistant TB still needs to improve, primarily due to the limited availability of effective TB medications and the growing challenge of drug resistance. Urgent action is imperative to meet the targets outlined in the End TB 2035 strategy, underscoring the critical need for the development of novel and efficacious anti-TB drugs (2–4).

In the treatment of drug-susceptible tuberculosis, first-line anti-TB drugs alone cannot meet the therapeutic needs. Thus, fluoroquinolones often play an essential role. Fluoroquinolones such as levofloxacin and moxifloxacin demonstrate potent antitubercular activity by inhibiting DNA gyrase, thereby impeding normal DNA replication, transcription, transport, and recombination (5). Levofloxacin and moxifloxacin are categorized by the WHO as group A drugs for treating drug-resistant tuberculosis, due to their robust bactericidal effects. However, the emergence of fluoroquinolone resistance, usually caused by primary resistance or irrational use, is common in clinical settings, thereby restricting their use (6–8). Furthermore, innovative treatment protocols for fluoroquinolone-resistant tuberculosis, exemplified by regimens like bedaquiline, pretomanid, and linezolid, have notably curtailed the duration of treatment and bolstered the rates of successful outcomes (4). However, it is a stark reality that a subset of patients remains ineligible for these advanced therapies because of a myriad of factors. Consequently, the imperative for research into novel fluoroquinolones, including sitafloxacin, in tuberculosis treatment is both pressing and paramount.

Sitafloxacin, belonging to the new generation of broad-spectrum fluoroquinolone antimicrobials, features a cis-fluorocyclopropylamine group in its structure, endowing it with favorable pharmacokinetic properties. Notably, it exhibits enhanced antimicrobial efficacy compared to other quinolones against a spectrum of pathogens, including Gram-positive, Gram-negative, and atypical bacteria, along with various drug-resistant strains, while also mitigating adverse effects (9–11). Moreover, sitafloxacin demonstrates potent inhibition of the quinolone resistance-determining region mutase activity, reducing the likelihood of resistance development compared to other quinolone antimicrobials (12, 13). In pharmacological and *in vitro* studies, sitafloxacin displays robust bactericidal activity against *Mycobacterium tuberculosis*, including drug-resistant strains, surpassing moxifloxacin, levofloxacin, and ciprofloxacin (13). To elucidate sitafloxacin’s therapeutic role and safety in primary tuberculosis treatment and lay a foundation for further exploration, we investigated its early bactericidal activity in patients undergoing initial tuberculosis treatment.

## MATERIALS AND METHODS

### Study design and participants

The study employed a phase two randomized, open-label, multicenter, positively controlled clinical trial design (ClinicalTrials.gov identifier MR-11-23-024134). Its primary objective was to assess the early bactericidal activity of sitafloxacin in patients diagnosed with primary tuberculosis. Thirty participants, aged between 18 and 60 years, were enrolled in three medical centers: Beijing Chest Hospital affiliated with Capital Medical University, Chengdu Public Health Clinical Medical Center, and Wuhan Lung Hospital. Inclusion criteria stipulated patients with positive sputum smear(≥2+), confirmed to have *Mycobacterium tuberculosis* via the Xpert assay, and showing drug-susceptible to rifampicin. Additionally, participants were required to test negative for isoniazid and fluoroquinolone resistance genes and have no history of prior antituberculosis treatment.

### Study procedures

Participants were randomly assigned to one of three groups: the INH group, the levofloxacin group, and the sitafloxacin group. Each group received oral medication once daily for 7 consecutive days: INH 300 mg, levofloxacin 500 mg, and sitafloxacin 200 mg. The administration of the study drug was directly observed at the enrollment hospital, with overnight sputum specimens collected from all subjects for 16 hours daily. The staff performing bacterial cultures were single-blinded to the treatment grouping of patients. Vital signs and physical examinations were performed daily throughout the study period. Safety assessments, including electrocardiogram (ECG), hematology, biochemistry, and urine parameter evaluations, were conducted on days 1, 3, and 7, at various time points.

### Mycobacteriology

Before randomization, sputum samples collected from subjects were analyzed for rifampicin susceptibility and genotypic identification using the GeneXpert MTB/RIF assay (American Cepheid Company). Additionally, baseline cultures were conducted using the BD Bactec MGIT 960 mycobacterium culture system (Becton Dickinson). Culture-positive samples were tested to determine phenotypic susceptibility to isoniazid and quinolones. To assess bactericidal activity, overnight sputum samples were collected from each subject for 16 consecutive hours (from 16:00 to 08:00), yielding a minimum of 5 mL. All sputum specimens were processed within 2 hours of collection (14, 15).

For processing, the mixed sputums were homogenized with 1% dithiothreitol and vortexed for 20 seconds. The mixture was then placed on a rocker at 60 rpm for 20 minutes. Subsequently, 10-fold dilutions ranging from 100 to 10^−5^ were prepared by aspirating 100 µL of the digested sample. Each dilution was inoculated in triplicate onto Middlebrook 7H11 agar culture plates supplemented with 10% OADC (oleic acid-albumin-bovine-dextrose-catalase) and four selective antibiotics (polymyxin B, amphotericin B, carbenicillin, and methicillin). The plates were homogeneously coated and then incubated in a 37°C, 5% CO2 environment for 4–6 weeks. Colonies ranging from 20 to 200 visible colonies were counted. Where accurate counting was not feasible within this range, then as many colonies as possible were counted. The counts were averaged and adjusted for dilution to determine the CFU per milliliter of sputum (14).

### Statistical analyses

The CFU results collected from all participants were analyzed to assess the treatment effect by measuring the change in log_10_CFU/mL of sputum from baseline. This change was expressed as the mean ± standard deviation of the day-to-day decrease in log_10_CFU/mL. log_10_CFU counts were set at 0 when CFU counts were zero. The primary treatment endpoint was the EBA effect over a specific period (EBA [0–7]), with secondary treatment endpoints including the EBA (0–2) and the EBA (2–7). These were calculated as EBA CFU = [log_10_CFU (day 0) − log_10_CFU (day X)] / (0−X), expressed as the reduced value of log_10_CFU·mL^−1^·d^−1^. Furthermore, the rate of decline in sputum CFU from days 2–7 (b2-7) was estimated using the slope of a linear regression derived from CFU values of the six sputum specimens collected on days 2–7 of interest (16, 17). All statistical analyses were conducted at a two-tailed 5% significance level. Treatment effects were compared with the control group using either one-way analysis of variance (ANOVA) or the Kruskal–Wallis test. Statistical analysis was performed using SPSS 21.0, with a *P* value <0.05 considered statistically significant.

## RESULTS

### Study population

Thirty subjects, aged between 18 and 60 years, were recruited from the Beijing Chest Hospital of Capital Medical University, Chengdu Public Health Clinical Medical Center, and Wuhan Lung Hospital. All participants had positive sputum smears (≥2+). They were confirmed to have *Mycobacterium tuberculosis* and were diagnosed with primary sensitive tuberculosis. None of the participants had received prior antituberculosis medication. The distribution of subjects among the three groups was similar in terms of demographic characteristics such as age, sex, height, weight, and BMI. Additionally, all subjects tested negative for HIV and exhibited comparable baseline characteristics (see Table 1).

**TABLE 1:** Demographics and baseline characteristics—all randomized subjects^*a*^

	INH 300 mg (*N*=8) n (%)	Levofloxacin 500 mg (*N*=10) n (%)	Sitafloxacin 200 mg (*N*=12) n (%)	*P* value
Age (years)				
Median	37.1	32.2	36.3	0.69
IQR	24.3, 51.0	20.8, 44.3	25.3, 51.0	
Gender				0.41
Male	7 (87.5)	7 (70.0)	7 (58.3)	
Female	1 (12.5)	3 (30.0)	5 (41.7)	
Height (cm)				
Median	167.8	169.9	167.6	0.72
IQR	161.3, 173.0	162.8, 175.0	163.3, 172.3	
Weight (kg)				
Median	54.9	62.2	58.7	0.21
IQR	50.25, 59.0	51.8, 71.3	50.8, 65.0	
BMI (kg/m^2^)				
Median	19.5	21.4	20.9	0.27
IQR	18.2, 20.7	19.6, 24.3	19.1, 22.5	
Country				
China	8 (100.0)	10 (100.0)	12 (100.0)	
HIV				
Positive	0	0	0	
Negative	8 (100.0)	10 (100.0)	12 (100.0)	
Baseline CFU (log_10_/mL sputum)	5.65 (0.85)	4.90 (1.50)	5.98 (0.82)	0.086

^*a*^ INH, isoniazid; IQR, interquartile range; BMI, body mass index; HIV, human immunodeficiency virus.

### Bactericidal activity

The primary treatment endpoint for assessing bactericidal activity was the EBA measured from days 0 to 7. Secondary endpoints included EBA from days 0 to 2 and from days 2 to 7. Differences in the mean daily decline in the number of viable bacteria after drug administration were evaluated for each patient group using one-way ANOVA. Additionally, the primary endpoint, daily viable bacteria counts, was analyzed utilizing a mixed-effects model based on repeated measurement information. This involved calculating the mean change in each treatment group at each visit and estimating the point difference in mean change between the treatment groups along with a two-sided 95% confidence interval (95% CI).

**Fig 1:**
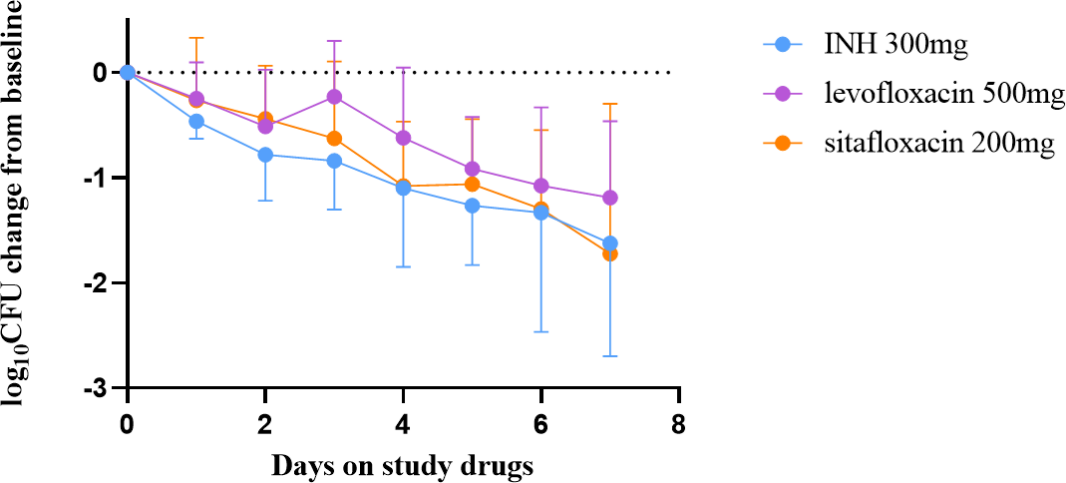
Change in colony-forming units (CFU) in sputum before and during 7 days of study drug administration with isoniazid (INH, 300 mg once daily)、levofloxacin（ 500 mg, once daily）and sitafloxacin(200 mg, once daily). Sputum was collected for 16 hours for 2 days before and daily during 7 days of drug administration. Data represent the mean change in log10 CFU/ml of sputum ± SD for each of the 7 days of study drug administration. Mean baseline colony-forming unit counts for each treatment group are listed in the text.

The mean baseline concentration of Mtb per milliliter of sputum in INH, levofloxacin, and sitafloxacin groups (log_10_CFU/mL mean ± standard deviation) was 5.65 ± 0.85, 4.90 ± 1.50, and 5.98 ± 0.82, respectively, and did not differ between each group. The daily decline in sputum viable bacteria is shown in Fig. 1. There was a decline in the number of sputum viable bacteria in every group over the 7 days. The decline in sputum viable bacteria in the sitafloxacin showed the same decline as the INH group on days 4 and 6 and exceeded the isoniazid group on day 7, without statistical significance(*P* = 0.53). Significantly, the decreased rate in the sitafloxacin group was almost always higher than in the levofloxacin group. The slopes of decline in the secondary treatment endpoints EBA 0–2 and EBA 2–7 and the rate of decline in sputum CFU from days 2–7 (b2-7) were analyzed in Tables 2 and 3, respectively. The EBA 0–2 of INH (0.39 ± 0.22) was higher than levofloxacin (0.26 ± 0.27) and sitafloxacin (0.22 ± 0.25), which was not statistically different (*P* = 0.08), showing similar EBA of sitafloxacin with INH and levofloxacin. INH EBA2-7 (0.17 ± 0.16) was higher than levofloxacin (0.14 ± 0.10), but both were lower than sitafloxacin (0.26 ± 0.31), with no statistical difference (*P* = 0.59). The results of the slope of the decline of sputum CFU on days 2–7 (b2-7) also showed that the sitafloxacin group has an extended EBA than the INH and levofloxacin groups. Although not statistically significant, sitafloxacin had a higher decline rate than INH and levofloxacin.

**TABLE 2:** Early bactericidal activity: days 0 to 2^*a*^

Drug	n	Mean EBA (log_10_ CFU/mL/day)	SD	95% CI
INH	8	0.39	0.22	0.24–0.53
Levofloxacin	10	0.26	0.27	0.09–0.43
Sitafloxacin	12	0.22	0.25	0.08–0.38

^*a*^ CFU, colony-forming units; EBA, early bactericidal activity; INH, isoniazid; 95% CI, 95% confidence interval; SD, standard deviation. *P* ＞ 0.05 compared to INH.

**TABLE 3:** Extended early bactericidal activity: days 2 to 7^*a*^

Drug	n	Mean EBA (log_10_ CFU/mL/day)	SD	95% CI	Mean slope of CFU between days 2 and 7; b2-7 (log_10_ CFU/mL/day)	SD	95% CI
INH	8	0.17	0.16	0.05–0.28	0.18	0.18	0.02–0.32
Levofloxacin	10	0.14	0.10	0.07–0.20	0.17	0.13	0.07–0.27
Sitafloxacin	12	0.26	0.31	0.10–0.44	0.22	0.26	0.05–0.38

^*a*^ CFU, colony-forming units; EBA, early bactericidal activity; INH, isoniazid; 95% CI, 95% confidence interval; SD, standard deviation. *P* ＞ 0.05 compared to INH.

Concurrently, we conducted an analysis of the early bactericidal activity (EBA0-3, EBA0-5, and EBA0-7) of the three groups (refer to Table 4). Our findings revealed that the EBA0-3 of the levofloxacin group was lower than that of the INH group, displaying a statistically significant difference. This indicates that levofloxacin exhibited less bactericidal activity than INH during the initial 3 days of treatment. Sitafloxacin demonstrated EBA values lower than isoniazid but higher than levofloxacin at both EBA0-3 and EBA0-5. Furthermore, sitafloxacin exhibited higher EBA values than both INH and levofloxacin at EBA0-7, although the difference was not statistically significant. These findings suggest that sitafloxacin maintained better bactericidal activity throughout the treatment duration compared to both INH and levofloxacin.

**TABLE 4:** Extended early bactericidal activity: days 0 to 3, 0 to 5, and 0 to 7^*b*^

Drug	n	Days 0 to 3 Mean EBA (log_10_ CFU/mL/day)	SD	95% CI	Days 0 to 5 Mean EBA (log_10_ CFU/mL/day)	SD	95% CI	Days 0 to 7 Mean EBA (log_10_ CFU/mL/day)	SD	95% CI
INH	8	0.28	0.16	0.17–0.38	0.25	0.11	0.18–0.32	0.23	0.15	0.13–0.33
Levofloxacin	10	0.08^*a*^	0.18	−0.02–0.20	0.18	0.10	0.12–0.25	0.17	0.10	0.11–0.23
Sitafloxacin	12	0.21	0.24	0.08–0.35	0.21	0.12	0.14–0.28	0.24	0.20	0.10–0.44

^*a*^ *P* ＜ 0.05 compared with INH.

^*b*^ CFU, colony-forming units; EBA, early bactericidal activity; INH, isoniazid; 95% CI, 95% confidence interval; SD, standard deviation.

### Safety

Throughout the study duration, all 30 participants successfully completed the treatment regimen without experiencing any serious adverse events such as leukopenia, anemia, severe liver function damage (aminotransferase is 10 times or more above the upper limit of normal value), renal injury (serum creatinine over 2 mg/dL), or ECG abnormalities (the ECG QT interval over 500 ms) according to the relevant examinations on the third and the end of treatment. However, one participant in each group developed a fever during the treatment period, attributed to tuberculosis infection, and their symptoms were alleviated following appropriate symptomatic management including antipyretic therapy. In the INH group, one subject exhibited weakly positive urinary protein during the final safety monitoring, which normalized during subsequent follow-up assessments. In the sitafloxacin group, one participant experienced a single episode of vomiting due to coughing, a common symptom of tuberculosis, with no further episodes reported. Notably, all subjects successfully completed the standard antituberculosis therapy regimen (2‌Isoniazid-Rifampicin-Pyrazinamide-Ethambutol/4Isoniazid-Rifampicin, 2HRZE/4HR). Importantly, during the study and subsequent anti-TB treatment, no subjects developed resistance to isoniazid, rifampicin, or levofloxacin by sputum examination.

## DISCUSSION

To gain deeper insights into the bactericidal activity and safety profile of sitafloxacin, this study focused on patients diagnosed with primary drug-susceptible tuberculosis. These patients were randomly allocated into isoniazid, levofloxacin, and sitafloxacin groups for a 7-day monotherapy regimen. Analysis of the data revealed a sequential decrease in EBA0-2 across the isoniazid, levofloxacin, and sitafloxacin groups, consistent with findings from previous studies. Notably, the isoniazid group, serving as the positive control, exhibited the highest EBA0-2. Conversely, sitafloxacin demonstrated a slightly lower EBA compared to levofloxacin, with no statistically significant difference observed among the three groups. These findings suggest that sitafloxacin exhibits comparable EBA to both INH and levofloxacin, indicating its ability to swiftly penetrate tuberculosis lesions and eradicate fast-growing bacilli (11, 18).

The slope of decline in sputum CFU from day 2 to day 7 (b2-7) indicates that sitafloxacin exhibits a prolonged, higher extended early bactericidal activity compared to isoniazid and levofloxacin. Additional analysis of EBA at day 3, day 5, and day 7 (EBA0-3, EBA0-5, and EBA0-7) across the three groups revealed that sitafloxacin had lower EBA values than isoniazid at day 3 and day 5 but higher values than levofloxacin at these time points. By day 7, sitafloxacin demonstrated higher EBA values compared to both isoniazid and levofloxacin, though there was no statistical difference.

Moreover, the reduction in sputum viable bacteria in the sitafloxacin group paralleled that of the isoniazid group on days 4 and 6 and even exceeded it on day 7. Notably, the reduction in the sitafloxacin group consistently surpassed that in the levofloxacin group almost throughout the study duration. These findings suggest that sitafloxacin exhibits a higher prolonged early bactericidal activity with stronger antituberculosis efficacy than levofloxacin. This enhanced activity may be attributed to sitafloxacin’s stronger dual inhibitory effect on DNA gyrase and topoisomerase, whereas levofloxacin primarily acts through inhibition of bacterial DNA helicase activity (11, 19).

Fluoroquinolones play a crucial role in treating drug-resistant tuberculosis; however, their efficacy is limited by the emergence of drug resistance. In contrast, sitafloxacin offers a dual potent antimicrobial effect and is less prone to developing high levels of resistance, except in cases of simultaneous mutations in DNA polymerase and topoisomerase. Notably, modifications to the targets of DNA gyrase or topoisomerase do not significantly impact sitafloxacin susceptibility (9, 20). A previous study conducted in Thailand demonstrated that minimum inhibitory concentrations of sitafloxacin are notably lower than those of moxifloxacin, levofloxacin, and ciprofloxacin against drug-resistant tuberculosis, indicating potent *in vitro* activity (10, 13). Collectively, our preliminary *in vivo* evidence underscores the potential of sitafloxacin in the clinical management of both drug-susceptible and drug-resistant tuberculosis.

The findings of this study should be interpreted within the context of several limitations. Firstly, due to the considerable quantity of sputum specimens required and the logistical challenges associated with conducting an EBA study, blood concentrations of the subjects were not monitored during drug administration. However, based on relevant published studies (10, 18), sitafloxacin has been shown to have a lower MIC, and pharmacokinetics show rapid absorption after oral administration and good tissue/body fluid distribution. Secondly, the sample size in this study was limited, as it was a small cohort study. Consequently, there may be a limited ability to detect small differences in EBA0-2 and EBA2-7 between the study groups, despite efforts to recruit patients with high sputum bacterial loads to minimize variability. Lastly, although this study was randomized and open-label, with staff members aware of the medications each patient received, the primary endpoint assessed was bacteriology. Each enrolled individual was randomly assigned to a treatment group, and sputum specimens were anonymized with identification numbers. Additionally, laboratory staff responsible for quantitative cultures and drug susceptibility testing were single-blind to the treatment group allocation of the patients. Finally, levofloxacin exhibits a concentration-dependent effect against Mtb. The dosing in EBA studies is usually given as 750–1,000 mg/day. Although the dose of this study was 500 mg/day, it also showed a good bactericidal effect, which can be used as a control to observe the bactericidal of sitafloxacin.

In conclusion, sitafloxacin demonstrates comparable early bactericidal activity and extended early bactericidal activity compared to levofloxacin. Moreover, our findings indicate that this novel fluoroquinolone exhibits a favorable safety profile during the study period. Therefore, our data underscore the potential of sitafloxacin in the clinical management of both drug-susceptible and drug-resistant tuberculosis.

## Data Availability

The authors have the willingness to share all data used in this study upon reasonable request and application to the corresponding author, if the application is approved.

## SUPPLEMENTAL MATERIAL

Supplemental material spectrum.01645-24-s0001.docx Graphical abstract; highlights; Fig. S1.

ASM does not own the copyrights to Supplemental Material that may be linked to, or accessed through, an article. The authors have granted ASM a non-exclusive, world-wide license to publish the Supplemental Material files. Please contact the corresponding author directly for reuse.
