# Supplementary material for: Early bactericidal activity of sitafloxacin against pulmonary tuberculosis
Source: Microbiol Spectr. 2024 Dec 10;13(1):e01645-24. doi: 10.1128/spectrum.01645-24 (PMC11705927; doi:10.1128/spectrum.01645-24)
Supplement: Supplemental material — Graphical abstract; highlights; Fig. S1. [file spectrum.01645-24-s0001.docx]

**Early bactericidal activity of sitafloxacin against pulmonary tuberculosis**

Lihui Nie^1^, Jing Tong^2^, Guihui Wu^3^, Juan Du^4^, Yuanyuan Shang^2^, Yufeng Wang^5^, Zhangjun Wu^3^, Yuanhong Xu^3^, Yi Ren^4^, Youyi Rao^4^, Yu Pang^2^, Mengqiu Gao^1^

**Graphical Abstract**


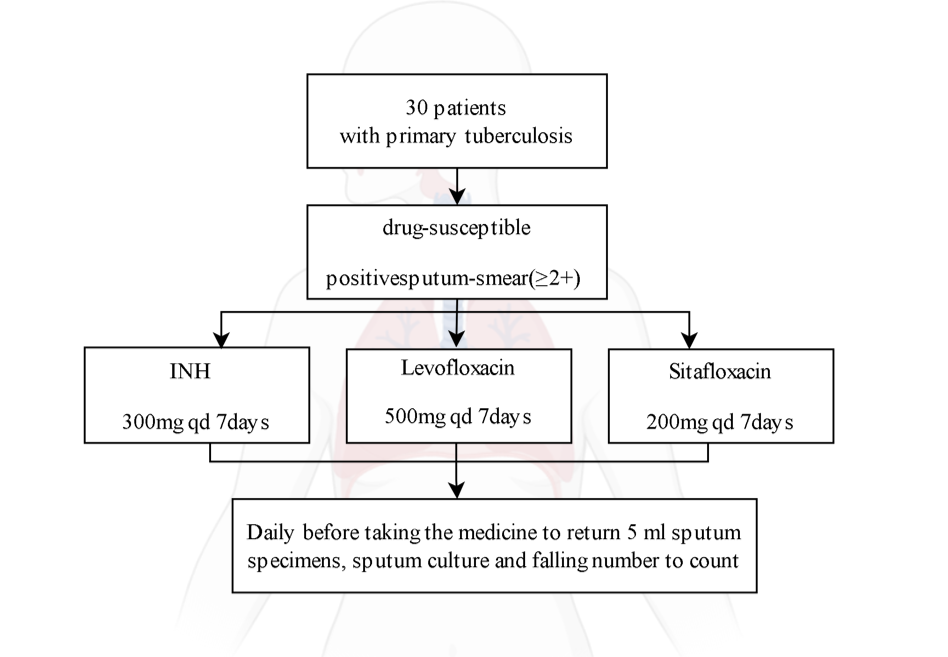


**Highlights**

1.The study investigated the early bactericidal activity of sitafloxacin in primary susceptible tuberculosis.

2. Sitafloxacin has good bactericidal activity.

3. Sitafloxacin will most likely be a core drug for the treatment of TB.

Figure S1. Change in colony-forming units (CFU) in sputum before and during 7 days of study drug administration with isoniazid (INH, 300 mg once daily)、levofloxacin（500 mg, once daily）and sitafloxacin (200 mg, once daily). Sputum was collected for 16 hours for 2 days before and daily during 7 days of drug administration. Data represent the mean change in log_10_ CFU/ml of sputum ± SD for each of the 7 days of study drug administration. Mean baseline colony-forming unit counts for each treatment group are listed in the text.
